# Dimensional Lifting through the Generalized Gram–Schmidt Process

**DOI:** 10.3390/e20040284

**Published:** 2018-04-14

**Authors:** Hans Havlicek, Karl Svozil

**Affiliations:** 1 Institute of Discrete Mathematics and Geometry, Vienna University of Technology, Wiedner Hauptstraße 8-10/104, A-1040 Vienna, Austria; 2 Institute for Theoretical Physics, Vienna University of Technology, Wiedner Hauptstraße 8-10/136, A-1040 Vienna, Austria; 3 Department of Computer Science, University of Auckland, Auckland 1142, New Zealand

**Keywords:** orthogonality, quantum computation, Gram–Schmidt process, 03.65.Aa, 02.10.Ud, 02.30.Sa, 03.67.Ac

## Abstract

A new way of orthogonalizing ensembles of vectors by “lifting” them to higher dimensions is introduced. This method can potentially be utilized for solving quantum decision and computing problems.

The celebrated Gram–Schmidt algorithm allows the construction of a system of *orthonormal* vectors from an (ordered) system of *linearly independent* vectors. Let us mention that there exist a wide variety of proposals to “generalize” the Gram–Schmidt process [1] serving many different purposes. In contrast to these generalizations, we construct a system of *orthogonal vectors* from an (ordered) system of *arbitrary* vectors, which may be linearly dependent. (Even repeated vectors are allowed.) This task is accomplished by what will be called “dimensional lifting”.

Some quantum computation tasks require the orthogonalization of previously non-orthogonal vectors. This might be best understood in terms of mutually exclusive outcomes of generalized beam splitter experiments, where the entire array of output ports corresponds to an ensemble of mutually orthogonal subspaces, or, equivalently, mutually orthogonal perpendicular projection operators [2].

Of course, by definition (we may define a unitary transformation in a complex Hilbert space by the requirement that it preserves the scalar product [3] (§ 73)), any transformation or mapping of non-orthogonal vectors into mutually orthogonal ones will be non-unitary. However, we may resort to requiring that some sort of angles or distances (e.g., in the original Hilbert space) remain unchanged.

Suppose, for the sake of demonstration, two non-orthogonal vectors, and suppose further that somehow one could “orthogonalize” them while at the same time retaining structural elements, such as the angles between projections of the new, mutually orthogonal vectors onto the subspace spanned by the original vectors. For instance, the two non-orthogonal vectors could be transformed into vectors of some higher-dimensional Hilbert space satisfying the following properties with respect to the original vectors: (i) the new vectors are orthogonal, and (ii) the orthogonal projection along the new, extra dimension(s) of the two vectors render the original vectors. A straightforward three-dimensional construction with the desired outcome can be given as follows: suppose the original vectors are unit vectors denoted by $|e_{1}\rangle$ and $|e_{2}\rangle$; and $0<|\langle e_{1}|e_{2}\rangle |<1$. Suppose further a two-dimensional coordinate frame in which $|e_{1}\rangle$ and $|e_{2}\rangle$ are planar; thus, we can write in terms of some orthonormal basis $|e_{1}\rangle =\left(\begin{array}{c} x_{1,1},x_{1,2} \end{array}\right)$ as well as $|e_{2}\rangle =\left(\begin{array}{c} x_{2,1},x_{2,2} \end{array}\right)$. Suppose we “enlarge” the vector space to include an additional dimension, and suppose a Cartesian basis system in that greater space that includes the two vectors of the old basis (and an additional unit vector that is orthogonal with respect to the original plane spanned by the original basis vectors).

Ad hoc, it is rather intuitive how two (not necessarily unit) vectors can be found that project onto the original vectors, and which are orthogonal: “create” a three-dimensional vector space with one extra dimension, assign a non-zero extra coordinate (such as 1) associated with this dimension for the first vector, and use the extra coordinate of the second vector for compensating any nonzero value of the scalar product of the two original vectors— in particular, whose coordinates with respect to the new basis are

(1) $$\begin{array}{cc} |f_{1}\rangle = & \left(\begin{array}{c} x_{1,1},x_{1,2},1 \end{array}\right),\\ |f_{2}\rangle = & \left(\begin{array}{c} x_{2,1},x_{2,2},-\left(x_{1,1}x_{2,1}+x_{1,2}x_{2,2}\right) \end{array}\right), \end{array}$$

which are orthogonal by construction.

It is not too difficult to find explicit constructions for the more general case of *k* vectors $|e_{1}\rangle ,\ldots ,|e_{k}\rangle$ in $R^{n}$ (cf. Ref. [2] for a rather inefficient method).

In the following, for the sake of construction, we shall embed $R^{n}$ into $R^{n+k}$, such that we fill all additional vector coordinates of $|e_{1}\rangle ,\ldots |e_{k}\rangle$ with zeroes. For the new, mutually orthogonal, vectors we make the following *Ansatz* by defining

(2) $$\begin{array}{cc} |f_{1}\rangle = & \left(\begin{array}{c} e_{1},1,0,\ldots ,0 \end{array}\right),\\ |f_{2}\rangle = & \left(\begin{array}{c} e_{2},x_{2,1},1,0,\ldots ,0 \end{array}\right),\\ \ldots & \\ |f_{k}\rangle = & \left(\begin{array}{c} e_{k},x_{k,1},x_{k,2},\ldots ,x_{k,k-1},1 \end{array}\right), \end{array}$$

with yet to be determined coordinates $x_{i,j}$. (The symbols $e_{i}$ stand for all the *n* coordinates of $|e_{1}\rangle$.)

The unit coordinates 1 ensure that the new vectors are linearly independent. By construction, the orthogonal projection of $|f_{i}\rangle$ onto $R^{n}$ renders $|e_{i}\rangle$ for all 1≤i≤k.

What remains is the recursive determination of the unknown coordinates $x_{i,j}$. Note that all $|f_{j}\rangle$ must satisfy the following relations: for j>1, orthogonality demands that $\langle f_{1}|f_{j}\rangle =0$, and therefore $\langle e_{1}|e_{j}\rangle +1\cdot x_{j,1}=0$, and therefore

(3) $$x_{j,1}=-\langle e_{1}|e_{j}\rangle .$$

In this way, all unknown coordinates $x_{2,1},\ldots ,x_{k,1}$ can be determined.

Similar constructions yield the remaining unknown coordinates in $|f_{2}\rangle ,\ldots ,|f_{k}\rangle$. For j>2, $\langle f_{2}|f_{j}\rangle =0$, and therefore $\langle e_{2}|e_{j}\rangle +x_{2,1}x_{j,1}+x_{j,2}=0$, yielding

(4) $$x_{j,2}=-\langle e_{2}|e_{j}\rangle -x_{2,1}x_{j,1}.$$

In this way, all unknown coordinates $x_{3,2},\ldots ,x_{k,2}$ can be determined.

This procedure is repeated until one arrives at j=k−1, and therefore at the orthogonality of $|f_{k-1}\rangle$ and $|f_{k}\rangle$, encoded by the condition $\langle f_{k-1}|f_{k}\rangle =0$, and hence

(5) $$\begin{array}{c} x_{k,k-1}=-\left(\langle e_{k-1}|e_{k}\rangle +\right.\\ \left.+x_{k-1,1}x_{k,1}+\cdots +x_{k-1,k-2}x_{k,k-2}\right). \end{array}$$

The approach has the advantage that, at each stage of the recursive construction, there is only a single unknown coordinate per equation. This situation is well known from Gaussian elimination. The *Ansatz* also works if one of the original vectors is the zero vector, and if some of the original vectors are equal.

The resulting system of orthogonal vectors is not the only solution of the initial problem—to find an orthogonal vector that projects onto the original ones—which can be explicitly demonstrated by multiplying all vectors $|f_{1}\rangle ,\ldots ,|f_{k}\rangle$ with the matrix

(6) $$\mathrm{diag}\left(\begin{array}{c} I_{n},cT \end{array}\right),$$

whereby $I_{n}$ stands for the *n*-dimensional unit matrix, *c* can be a real nonzero constant, and T is a *k*-dimensional orthogonal matrix. (For complex Hilbert space, the orthogonal matrix needs to be substituted by a unitary matrix, and by a complex constant c≠0.)

On the other hand, we may reinterpret our procedure as follows: Let $|e_{1}\rangle ,\ldots ,|e_{k}\rangle$ be a system of vectors in $R^{n}$, not necessarily spanning $R^{n}$, and not necessarily being linearly independent. (The ordering of the vectors in this system will be essential throughout.) We embed $R^{n}$ in $R^{n+k}$ as we did above and denote the orthogonal complement of $R^{n}$ by $C≅R^{k}$. Therefore, the first *n* coordinates of all vectors in *C* vanish, and $R^{n+k}$ can be represented by a direct sum $R^{n+k}=R^{n}⊕R^{k}$. Additionally, we choose some (ordered) orthonormal basis of *C*, say, $|g_{1}\rangle ,\ldots ,|g_{k}\rangle$.

Then, there is a *unique* system of orthogonal vectors $|f_{1}\rangle ,\ldots ,|f_{k}\rangle$ in $R^{n+k}$ such that the following conditions are satisfied:

1. For all 1≤i≤k, the orthogonal projection of $R^{n+k}$ onto $R^{n}$ sends $|f_{i}\rangle$ to $|e_{i}\rangle$.
2. The orthogonal projection of $R^{n+k}$ onto *C* sends $|f_{1}\rangle ,\ldots ,|f_{k}\rangle$ to some (ordered) basis of the subspace *C*. Applying the Gram–Schmidt process to this (ordered) basis gives the orthonormal basis $|g_{1}\rangle ,\ldots ,|g_{k}\rangle$.

Indeed, in our previous *Ansatz*, we tacitly assumed the orthonormal basis $|g_{1}\rangle ,\ldots ,|g_{k}\rangle$ of *C* to comprise the orthogonal projections of the last *k* vectors of the standard basis $|b_{1}\rangle ,\ldots ,|b_{n+k}\rangle$ of $R^{n+k}$ onto *C*. Condition 2 enforces the presence of all the 1s and 0s in Formula (2), since the Gram–Schmidt process, applied to the vectors

$$|f_{1}\rangle -|e_{1}\rangle ,\ldots ,|f_{k}\rangle -|e_{k}\rangle ,$$

has to result in $|b_{n+1}\rangle ,\ldots ,|b_{n+k}\rangle$. Notice that the usual Gram–Schmidt process gives merely an *orthogonal* basis, whose vectors can be normalized in a second step in order to obtain an *orthonormal* basis. In our setting, however, such a second step is not allowed. As we saw above, now Condition 1 guarantees that $|f_{1}\rangle ,\ldots ,|f_{k}\rangle$ are uniquely determined.

Besides uniqueness, this construction has the additional advantage that the dot product in $R^{n+k}$ “decays” into the sum of dot products in $R^{n}$ and in $R^{k}$: any basis vector $f_{i}\in R^{n+k}$ can be uniquely written as $f_{i}=e_{i}+h_{i}$, where $e_{i}$ and $h_{i}$ represent the projection of $f_{i}$ along $h_{i}$ onto the original subspace $R^{n}$, and the projection of $f_{i}$ along $e_{i}$ onto *C*, respectively. Since $e_{i}$ is orthogonal to $h_{i}$, for i≠j, $f_{i}\cdot f_{j}=e_{i}\cdot e_{j}+h_{i}\cdot h_{j}=0,$ and thus

(7) $$\begin{array}{c} e_{i}\cdot e_{j}=-h_{i}\cdot h_{j}. \end{array}$$

Let us, for the sake of a physical example, study configurations associated with decision problems that can be efficiently (that is, with some speedup with respect to purely classical means [2]) encoded quantum mechanically. The *inverse problem* is the projection of orthogonal systems of vectors onto lower dimensions. This method renders a system of non-orthogonal rays, also called *eutactic stars* [4,5,6,7,8], which can be effectively levied to mutually exclusive outcomes in a generalized beam splitter configurations [9,10] reflecting the higher dimensional Hilbert space.

One instance of such a quantum computation involving the reduction to ensembles of orthogonal vectors (and their associated span or projection operators) is the Deutsch–Jozsa algorithm, as reviewed in Ref. [2]. Another, somewhat contrived, problem can be constructed in three dimensions from a eutactic star

(8) $$\begin{array}{c} \frac{1}{\sqrt{3}}\left\{\left(\begin{array}{c} 1,1 \end{array}\right),\left(\begin{array}{c} \frac{1}{2}\left[\sqrt{3}i-1\right],\frac{1}{2}\left[-\sqrt{3}i-1\right] \end{array}\right),\right.\\ \left.\left(\begin{array}{c} \frac{1}{2}\left[-\sqrt{3}i-1\right],\frac{1}{2}\left[\sqrt{3}i-1\right] \end{array}\right)\right\}, \end{array}$$

which is the projection onto the plane formed by the first two coordinates of a three-dimensional orthormal basis

(9) $$\begin{array}{c} B_{3}=\frac{1}{\sqrt{3}}\left\{\left(\begin{array}{c} 1,1,1 \end{array}\right),\right.\\ \left(\begin{array}{c} \frac{1}{2}\left[\sqrt{3}i-1\right],\frac{1}{2}\left[-\sqrt{3}i-1\right],1 \end{array}\right),\\ \left.\left(\begin{array}{c} \frac{1}{2}\left[-\sqrt{3}i-1\right],\frac{1}{2}\left[\sqrt{3}i-1\right],1 \end{array}\right)\right\}, \end{array}$$

which, together with the Cartesian standard basis, forms a pair of unbiased bases [11].

Still another decision configuration is the eutactic star

(10) $$\begin{array}{c} \frac{1}{2}\left\{\left(\begin{array}{c} 1,1,1 \end{array}\right),\left(\begin{array}{c} 1,1,-1 \end{array}\right),\right.\\ \left(\begin{array}{c} 1,-1,1 \end{array}\right),\left.\left(\begin{array}{c} 1,-1,-1 \end{array}\right)\right\}, \end{array}$$

which is the projection onto the subspace formed by the first three coordinates of a four-dimensional orthormal basis

(11) $$\begin{array}{c} B_{4}=\frac{1}{2}\left\{\left(\begin{array}{c} 1,1,1,1 \end{array}\right),\left(\begin{array}{c} 1,1,-1,-1 \end{array}\right),\right.\\ \left.\left(\begin{array}{c} 1,-1,1,-1 \end{array}\right),\left(\begin{array}{c} 1,-1,-1,1 \end{array}\right)\right\}. \end{array}$$

More concretely, suppose some, admittedly construed, function *f*, and some quantum encoding |xf(y)〉, where *x* and *y* stand for (sequences of) auxiliary and input bits, respectively, would yield one of the basis systems $B_{3}$ or $B_{4}$. By reducing the auxiliary bits *x*, one might end up with the eutactic stars introduced above. Alas, so far, no candidate of this kind has been proposed.

In summary, a new method of orthogonalizing ensembles of vectors has been introduced. Thereby, the original vectors are “lifted” to or “completed” in higher dimensions. This method could be utilized for solving quantum decision and computing problems if the original problem does not allow an orthogonal encoding, and if extra bits can be introduced that render the equivalent of the extra dimensions in which the original state vectors can be lifted and orthogonalized.

Compared with methods that were introduced [12,13,14] previously to optimally differentiate between two non-orthogonal states, the scheme suggested here is similar in the sense that, in order to obtain a better resolution, the effective dimensionality of the problem is increased. However, our scheme is not limited to the differentiation between two states, as it uses arbitrary dimensionality. More importantly, whereas our scheme is capable of separating different states precisely, but in general is non-unitary—indeed, the original vectors are not mutually orthogonal, but the lifted vectors are, thereby changing the angles among vectors, resulting in transformations that cannot be unitary—the former methods are unitary and probabilistic.
